# TIPT2 and geminin interact with basal transcription factors to synergize in transcriptional regulation

**DOI:** 10.1186/1471-2091-10-16

**Published:** 2009-06-10

**Authors:** Mara E Pitulescu, Martin Teichmann, Lingfei Luo, Michael Kessel

**Affiliations:** 1 Research Group Developmental Biology, Department of Molecular Cell Biology, Max Planck Institute for Biophysical Chemistry, 37077 Göttingen, Germany; 2 Institut Européen de Chimie et Biologie (I.E.C.B.), Université Bordeaux 2 Victor Ségalen, INSERM U869, 2 rue Robert Escarpit, Pessac, F-33607, France

## Abstract

**Background:**

The re-replication inhibitor Geminin binds to several transcription factors including homeodomain proteins, and to members of the polycomb and the SWI/SNF complexes.

**Results:**

Here we describe the TATA-binding protein-like factor-interacting protein (TIPT) isoform 2, as a strong binding partner of Geminin. TIPT2 is widely expressed in mouse embryonic and adult tissues, residing both in cyto- and nucleoplasma, and enriched in the nucleolus. Like Geminin, also TIPT2 interacts with several polycomb factors, with the general transcription factor TBP (TATA box binding protein), and with the related protein TBPL1 (TRF2). TIPT2 synergizes with geminin and TBP in the activation of TATA box-containing promoters, and with TBPL1 and geminin in the activation of the TATA-less NF1 promoter. Geminin and TIPT2 were detected in the chromatin near TBP/TBPL1 binding sites.

**Conclusion:**

Together, our study introduces a novel transcriptional regulator and its function in cooperation with chromatin associated factors and the basal transcription machinery.

## Background

The core promoter is defined as the genomic region required for recruitment of the transcription apparatus and can be considered as the priming stage for transcription initiation [1]. Recruitment of RNA polymerase II requires the assembly of a preinitiation complex, including the basal transcription factors TFIIA, B, D, E, F and H near the transcriptional start site(s) [2]. In addition, transcriptional regulation often involves TAFs (TATA-associated factors), mediator complex(es), and positive or negative cofactors, which associate with cis-acting DNA sequences often located further upstream or sometimes downstream of the start site [1]. Only 10–20% of the promoters in mammals, Drosophila and Arabidopsis contain a TATA box, a conserved AT-rich sequence motif approximately 30 bp upstream from the first transcribed nucleotide [3-8]. In mammals, this minority of promoters is often associated with tissue-specific genes and high conservation across species [9,10]. The TATA box associates with TBP, the TATA box binding protein, which as an element of the TFIID factor also binds to many TATA-less promoters [11-14]. Genomic screening approaches in yeast suggest that the presence of TBP in the chromatin near a promoter does not necessarily correspond to transcriptional activity [15-17]. The adenovirus major late promoter (AdMLP) is a classical example for a TATA box promoter. It contains immediately upstream and downstream of the box additional protein binding sequences, the G-rich TFIIB recognition elements (BREs), which were also found in TATA-less promoters [18-21].

A TBP-like factor, TBPL1 (TLP/TLF/TRF2) has no affinity to the TATA box, but binds to other promoter elements, probably with a TBP-like function [22]. It was found in association with several promoters, e.g. of the *PCNA*, the *wee1*, the *neurofibromytosis type 1 *(*NF1*), the *histone H1*, and several ribosomal protein genes [23-30]. However, no common binding motif could so far be delineated in mammals or Drosophila.

Geminin is a cell cycle regulatory protein, which functions through an interaction with the replication licensing factor Cdt1 [31-33]. In addition, it was identified as a binding partner of developmental control proteins. It represses Hox functions during embryogenesis by direct association with Polycomb members on Hox regulatory chromatin elements, and by impairing Hox protein action on target genes through direct binding to the homeodomain [34,35]. In a similar way, geminin directly interacts and antagonizes the role of another homeobox protein, Six3 [36]. It represses the activator function of pro-neural basic helix-loop-helix transcription factors neurogenin 2 and NeuroD on their target neuron-specific genes by sequestering Brg1, the catalytic subunit of the chromatin remodeling complex SWI/SNF [37]. Alternatively, geminin represses neuronal gene transcription in non-neural cells through its association with the AP4 transcription factor, by recruiting the co-repressor SMRT and histone deacetylase HDAC3 to neuronal gene promoters [38].

Here, we describe the function of TIPT2, a protein we identified through its interaction with geminin. Both geminin and TIPT2 bind to the basic transcriptional machinery, and can activate transcription synergistically. They were found, alone or together, associated with the chromatin of specific promoters.

## Results

We previously identified the polycomb factor Scmh1 and the homeodomain proteins Hoxd10 and Hoxa11 as geminin binding partners in a yeast two-hybrid screen of a day 8.5 mouse embryo cDNA library [34]. In the same screen, a strongly interacting protein was identified, encoded by a 715 bp, poly(A)-tailed cDNA, corresponding to the Riken cDNA clone 5133400G04 [GenBank:NM_029485]. A database entry identified the protein as isoform 2 of "TIPT" (TATA-binding protein-like factor-interacting protein), more recently named TRF2 interacting protein in testis [GenBank:AAV97890] [39]. TIPT is encoded by three differentially spliced variants, TIPT isoform 1 [GenBank:AAV97890] (185 amino acids), TIPT isoform 2 [GenBank:AAH60950] (205 amino acids) and TIPT isoform 3 [GenBank:BAB30197](159 amino acids). Mammalian TIPTs are strongly conserved, often represented by several isoforms, but related proteins were not found in other vertebrates or Drosophila. There are no similarities to other known proteins, and no conserved domain was obvious. Two coiled-coil regions (amino acids 50–106 and 128–168), known to be protein-protein interaction sites, were predicted by the Simple Modular Architecture Research Tool program .

### TIPT2 expression

**Figure 1:**
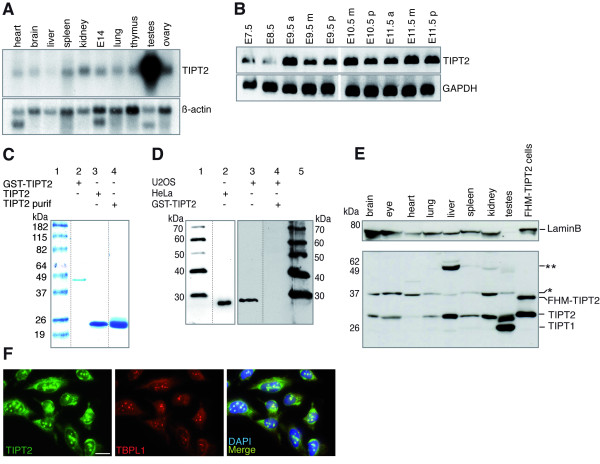
**RNA and protein expression of TIPT2**. (A) TIPT2 transcripts in mouse adult organs and embryonic (E14) tissues detected by Northern blotting. Beta actin was used as a loading control. (B) TIPT2 transcript in embryonic tissues prepared at the indicated stages of development detected by RT-PCR. a, m, and p RNAs were prepared from anterior, middle and posterior embryonic regions, respectively. (C) Bacterially made TIPT2 proteins. Lane 1: Marker. Lane 2: Coomassie blue stained recombinant GST-TIPT2. Lane 3: TIPT2, generated by thrombin cleavage, removal of GST by glutathione chromatography, and thrombin removal by benzamidine chromatography. Lane 4: Material equivalent to the protein in lane 3 was cut from a gel and electrophoresed again. (D) TIPT2 proteins detected by purified anti-TIPT antibodies in HeLa or U2OS whole cell extracts (lane 2 and 3). Detection of TIPT2 on the Western blot was competed by the inclusion of GST-TIPT2 in the antibody binding solution in a 10:1 ratio (lane 4). Markers were run on lanes 1 and 5. (E) TIPT2 proteins in organ extracts from P2 mice, adult mouse testis and a stable U2OS cell line expressing FHM-TIPT2. Asterisks indicate additional proteins recognized by anti-TIPT2 antibodies. (F) Cytoplasmic, nucleoplasmic and nucleolar localization of TIPT2 in human U2OS tissue culture cells. Nucleoli were detected with anti-TBPL1 antibodies. DNA was stained with DAPI. Bar, 20 μm. Further immunohistochemistry is presented in Additional file 1.

RNA from murine tissues was analyzed by Northern blotting. A transcript corresponding in length to isoform 2 of TIPT was found in all studied tissues (adult heart, brain, liver, spleen, kidney, lung, thymus, testes, ovary, and day 14 embryo), with particular abundance in testis (Figure 1A). RNA from younger embryonic stages was checked for the presence of TIPT2 mRNA by RT-PCR. Significant transcript levels were detected in day 7.5 and 8.5 embryos, and even more abundant levels in day 9.5, 10.5, and 11.5 embryos (Figure 1B). A polyclonal antibody was generated for further descriptive and functional analysis using for immunization the full-length mouse protein, cleaved from the purified *E.coli *recombinant GST-TIPT2 protein with thrombin, SDS-PAGE gel-purified, and verified by mass spectroscopy (Figure 1B). Immuno-purified antibodies detected the affinity-purified tagged and untagged bacterially expressed proteins (Figure 1C), and also the endogenous TIPT2 from HeLa or U2OS whole cell extract (Figure 1D). The detection of TIPT2 on Western blots could be blocked by GST-TIPT2 (Figure 1D). The major protein band detected by anti-TIPT2 antibodies in unfractionated extracts from HeLa, U2OS and other cells, and in organ extracts from newborn mice migrated slightly slower than the calculated size of 23,500 Da (Figure 1D, E, data not shown). The anti-TIPT2 antibody detected a larger sized protein band on some Western blots of gel purified TIPT2, and of protein extracts generated from mouse organs (Figure 1E, data not shown). After transfection of plasmids encoding tagged versions of TIPT2 the same bands were detected by anti-TIPT2 or anti-Tag antibodies (data not shown). After immunoprecipitation of HA-TIPT2 with HA antibodies the same pattern was detected by either anti-HA or anti-TIPT2 antibodies, and mass spectroscopy indicated HA-TIPT2 as the major precipitated protein (data not shown).

In mouse embryonic stem cells, SNL and NIH 3T3 mouse fibroblasts, monkey Cos-7, RD rat rabdomyosarcoma, HeLa human cervical cancer, and U2OS human osteosarcoma cells TIPT2 was localized both in cytoplasm and nucleus, with particular enrichment in nucleoli (Figure 1F, Additional file 1, data not shown). There, it co-localized with nucleolar markers, such as the basal transcription factor TBPL1, nucleophosmin (NPM), and the 15.5 K protein (Snu13p in yeast) [40-42]. HA-tagged TIPT2 co-localized in nucleoli and nucleoplasma with GFP-TBP after co-transfection of expression vectors (Additional file 1).

### TIPT2 interacting proteins

**Figure 2:**
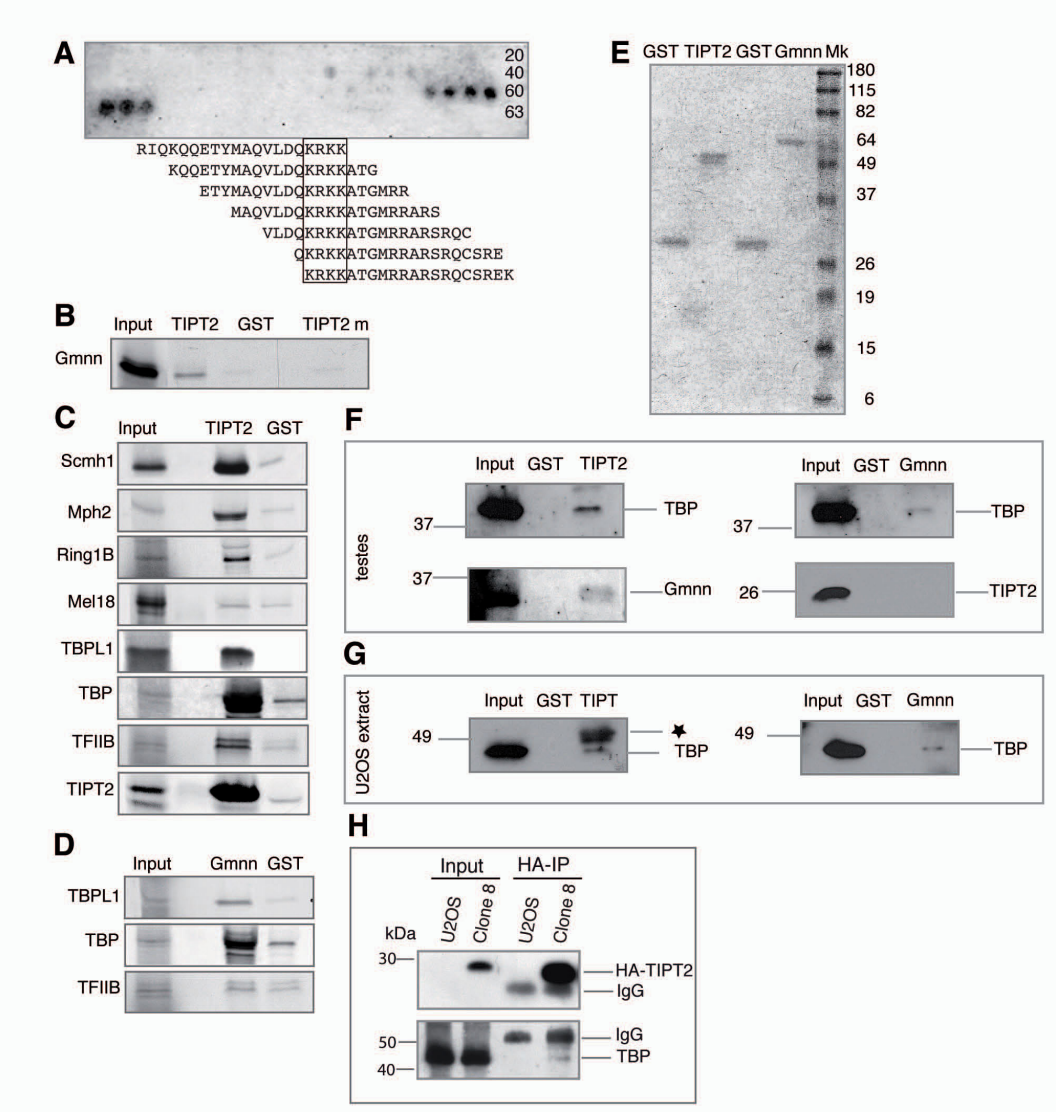
**Interaction partners of TIPT2 and geminin (Gmnn)**. (A) Binding of His-geminin to an array of 63 peptides with 17 amino acids overlapping 20-mers derived from the TIPT2 sequence. TIPT2 peptides bound specifically to His-geminin as detected by anti-geminin antibodies. KRKK amino acids of TIPT2 were mapped as the geminin-binding domain. (B-G) *In vitro *pull-down assays were performed using purified GST-TIPT2, GST-TIPT2m (containing a mutated geminin-binding site), GST-Gmnn or GST as bait. As preys were used *in vitro *transcribed/translated proteins (B-D), mouse adult testis (F) and human U2OS cell extracts (G). For experimental details please see methods section. (B) GST-TIPT2, GST-TIPT2m or GST interaction with radioactively labelled geminin. (C) GST-TIPT2 binding to *in vitro *translated Scmh1, Mph2, Ring1B, TBPL1, TBP, TFIIB, TIPT2 and Mel18. (D) GST-Gmnn interaction with *in vitro *translated TBP, TBPL1 and TFIIB. (E) Ponceau staining of GST proteins used for the protein lysate pull-down assays. (F) GST-TIPT2 and GST-Gmnn interaction with endogenous TBP, geminin and TIPT2 from adult mouse testis extract. (G) GST-TIPT2 and GST-Gmnn interaction with endogenous TBP from U2OS cell extract. The star indicates the cross-reactivity of TBP antibodies raised against GST recombinant human TBP with GST-TIPT2. (H) Co-immunoprecipitation of endogenous TBP with HA-TIPT2 using anti-HA antibodies. Extracts from untransfected U2OS cells, and from a U2OS clone stably producing HA-TIPT2 (HA-TIPT8), respectively, were analyzed.

The identification of TIPT2 in a yeast two-hybrid screen using geminin as bait prompted the investigation of an interaction with several candidates found in the chromatin or near promoters. *In vitro *pull-down assays were used to ensure that TIPT2 interacts directly with geminin (Figure 2B). To better characterize this interaction, the geminin binding site for TIPT2 was delineated by peptide array mapping. Binding of His-geminin to TIPT2 peptides revealed a basic amino acid rich region, close to the C-terminus of protein (KRKK, amino acids 185–189; Figure 2A). Indeed, a mutated GST-TIPT2 protein (TIPT2m; KRKK to DRDK) did not bind *in vitro *translated geminin (Figure 2B). Since geminin interacts with polycomb proteins, we checked also TIPT2 for a potential interaction. *In vitro *pull-down assays indicated the binding of Scmh1, Mph2, and Ring1B, but not of Mel18 (Figure 2C). In addition, the interaction of TIPT2 with itself was demonstrated *in vitro*. The nucleolar localization of TIPT2 and TBPL1, and the above mentioned database entry prompted us to analyze a possible interaction of TIPT2 with factors of the basal transcription machinery. *In vitro*, TIPT2 interacted directly with both TBP and TBPL1, and with TFIIB (Figure 2C). Because geminin binds TIPT2 and the latter binds basal transcription factors, geminin's interaction with TBP and TBPL1 was explored. The pull-down assay showed binding to both TBP family members, but not to TFIIB (Figure 2D). Further assays were performed in order to demonstrate the interaction of bacterially synthesized GST fusion proteins with endogenous proteins of cell lysates. Ponceau staining indicated equal inputs of GST proteins (Figure 2E). From an adult testis extract, both TBP and Geminin could be pulled-down with GST-TIPT2 (Figure 2F), and endogenous TBP could also be bound by GST-Geminin. However, endogenous TIPT2 could not be pulled-down by GST-Geminin, possibly because of an inhibitory effect of the GST tag. TBP in cell lysates prepared from human U2OS cells was pulled-down by both GST-TIPT2 and GST-Geminin (Figure 2G). In order to study the *in vivo *interaction of TIPT2 and TBP transfected cells were analyzed by co-immunoprecipitation. Anti-HA antibodies precipitated HA-TIPT2 together with endogenous TBP from a stable, HA-TIPT2 expressing cell line (HA-TIPT8; Figure 2H). In summary, we provide evidence that both TIPT2 and geminin bind members of the polycomb complex as well as basal transcription factors.

### TIPT2 and TBP bind to separate DNA elements

**Figure 3:**
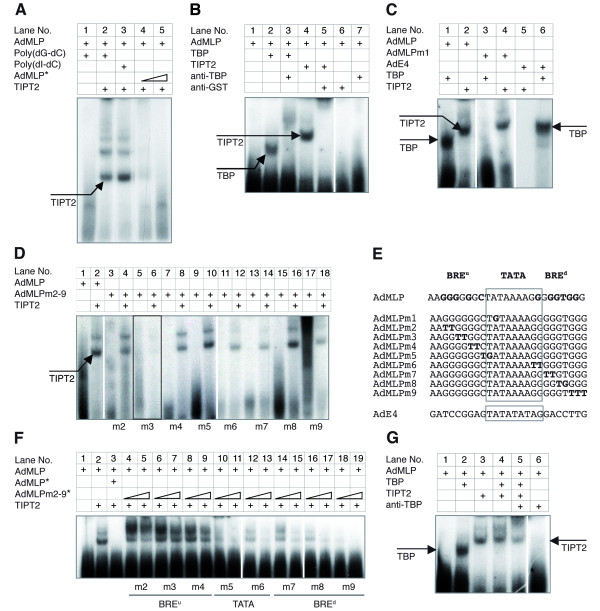
**Binding of TIPT2 and/or TBP to TATA box-containing promoters**. (A) Analysis of GST-TIPT2 binding to AdMLP. The specificity of the DNA-binding activity of 30 ng GST-TIPT2 was verified by competition with cold poly(dG-dC), poly(dI-dC), and unlabeled (*) AdMLP oligonucleotides, respectively. (B) The specificity of TBP and TIPT2 binding to AdMLP was tested by antibodies competition. Lane 2: The binding of 6 ng TBP to AdMLP. Lane 3: TBP-AdMLP complex was competed by anti-TBP antibodies. Lane 4: The binding of 15 ng GST-TIPT2 to AdMLP oligonucleotides. Lane 5: TIPT2-AdMLP complex was competed by anti-GST antibodies. (C) The binding of human TBP (6 ng) or GST-TIPT2 (30 ng) to AdMLP (containing BRE elements and a TATA box), AdMLPm1 (TATA box mutated) and AdE4 (no BRE elements). For sequences see Figure 3E. (D) GST-TIPT2 binding to AdMLP and m2–m9 mutants. (E) Oligonucleotide sequences of AdMLP representing AdMLP (BRE elements in bold), nine mutations of AdMLP (mutated bases highlighted), and AdE4. The TATA box motifs are framed. (F) Competition of the interaction between AdMLP and TIPT2 by mutated versions of AdMLP. Unlabelled oligonucleotides were preincubated with 6 ng GST-TIPT2. For sequences see Figure 3E. (G) Simultaneous binding of TIPT2 (60 ng) and TBP (6 ng) to AdMLP. The data in Figure 3B, D, F, G, respectively, stem from the same gel and exposure, the assembly is indicated by white lines.

Next, we analyzed whether TIPT2 interacts with promoter sequences. In a gel shift assay, TIPT2 bound strongly to the TATA box-containing AdMLP (Figure 3A, lanes 2,3). The interaction was highly specific and could be competed by cold AdMLP oligonucleotides, but not by high amounts of non-specific competitors (Figure 3A). Multiple bands were detected in the gel shift assay, suggesting that TIPT2 may not only interact with DNA as a monomer, but in addition more than one TIPT2 monomer or a multimer may bind to the AdMLP oligonucleotide. The migration of TBP or GST-TIPT2 bound proteins to AdMLP was retarded when anti-TBP, respectively anti-GST monoclonal antibodies were added to the binding reactions (Figure 3B, lanes 3,5). An AdMLP oligonucleotide with a mutated TATA box (AdMLPm1; Figure 3E) was still recognized by TIPT2, but not by human TBP (Figure 3C, lanes 3,4). TIPT2 did not bind to an oligonucleotide representing the AdE4 promoter (Figure 3E) [43], to which TBP bound strongly (Figure 3C, lanes 5,6). These data indicate that TIPT2 does not interact with TATA box sequences, and TBP requires a bona fide TATA box. The AdMLP contains G-rich BRE elements flanking the TATA box [18,21]. To reveal the location of the TIPT2 binding site, sequential mutations were introduced into the BRE elements of AdMLP (Figure 3E). The binding was impaired only for AdMLPm3, where the central GG sequence of the BRE upstream (BRE^u^) element was substituted by TT (Figure 3D, lanes 5,6; 3E). In contrary, the mutations AdMLPm2 and m4–m9 still bound directly to TIPT2 (Figure 3D, lanes 3,4 and 7–18). The binding of TIPT2 to AdMLP was competed with the cold oligonucleotides AdMLPm2–m9. Mutants of the BRE^u ^element (m2, m3, m4) did not compete for AdMLP binding (Figure 3F, lanes 4–9), whereas mutations in the TATA box (m5, m6) or in the BRE^d ^element (m7, m8, m9) competed well for AdMLP binding (Figure 3F, lanes 10–19). Together, these findings emphasized the importance of the BRE^u ^element, in particular its center position, for the binding of TIPT2. In this context potential interaction partners of TFIIB were explored. In a pull-down assay we observed that TIPT2, but not geminin associated with TFIIB (Figure 2C, D).

TBP retarded significantly AdMLP oligonucleotides, which were clearly separated from TIPT2/AdMLP bands (Figure 3G, lanes 2,3). The TBP/AdMLP band completely disappeared when both TBP and TIPT2 were present in the binding reaction, while the TIPT2/AdMLP complex was maintained (Figure 3G, lane 4). More retarded oligonucleotides were observed above the TIPT2/AdMLP DNA complex, which may indicate the formation of a ternary TIPT2/TBP/DNA complex, but could not be defined in a clear band. When anti-TBP antibodies were added to this binding reaction, the TIPT2/DNA complex was still present, and even more retarded oligonucleotides were observed (Figure 3G, lane 5), possibly indicating that TIPT2 and TBP bind independently of each other to the AdMLP.

### TIPT2, TBP and geminin synergize to activate the TATA box-containing promoters

**Figure 4:**
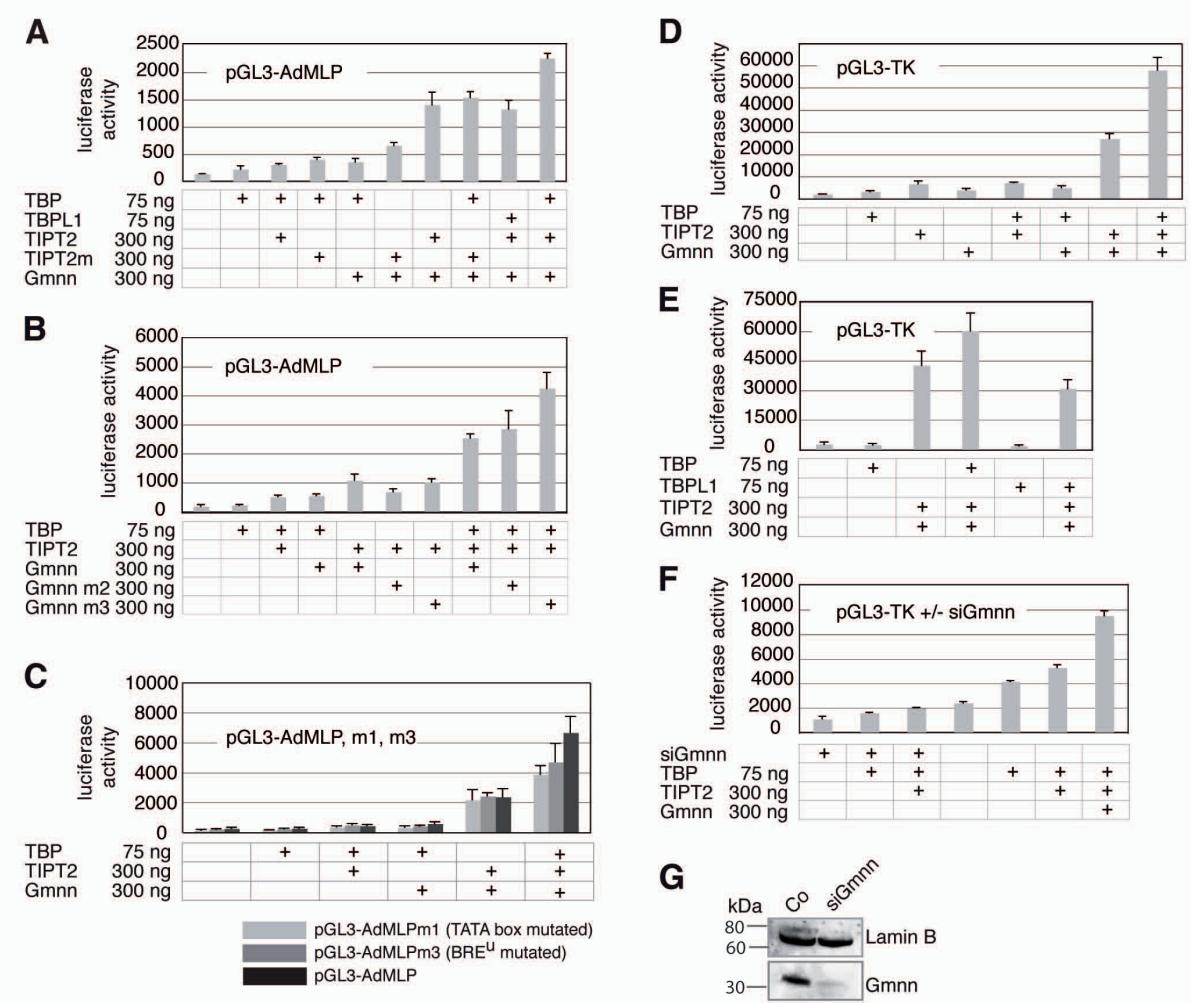
**Geminin cooperates with TIPT2 and TBP to activate TATA box-containing promoters**. (A) Activation of the AdMLP by the indicated combinations of TIPT2, TIPT2m, TBP, TBPL1 and geminin. (B) Activation of the AdMLP by TBP, TIPT2, and geminin or geminin mutants. Gmnn m2 lacks the coiled-coil domain, Gmnn m3 lacks the Brg1 binding domain. (C) Activation of the reporters pGL3-AdMLP, pGL3-AdMLPm1 (mutated TATA box), and pGL3-AdMLPm3 (mutated BRE^u^element) by the indicated combinations of TBP, TIPT2, and geminin. For sequence motifs see Figure 3E. (D) Activation of the TK promoter by the indicated combinations of TBP, TIPT2, and geminin. (E) Activation of the TK promoter by the indicated combinations of TBP, TBPL1, TIPT2, and geminin. (F) Activation of the TK promoter by the indicated combinations of TBP, TIPT2, and geminin, in the presence of siRNA against geminin. (G) Knock-down of endogenous geminin protein by transfection of U2OS cells with siRNA. The level of Lamin B protein indicates the gel loading of the protein extracts.

We next performed transient luciferase reporter assays testing the influence of TIPT2, TBP and/or geminin on the AdML promoter. Vector encoded proteins were present in amounts similar to endogenous proteins, as demonstrated by Western blots for transiently transfected GFP-TIPT2 and FHM-TIPT2 (data not shown). Transfecting in addition to TBP also TIPT2, TIPT2m (containing a mutated geminin binding site) or geminin enhanced the activation of the reporter only marginally (2–3 fold; Figure 4A). In comparison, the co-transfection of geminin with TIPT2, but not TIPT2m (p-value < 0,001), was much more effective (11 fold). An optimal activation level (16 fold) was observed in triple transfections, when TBP, TIPT2 and geminin were introduced together. This effect was attenuated when TIPT2m instead of TIPT2 (p-value < 0,001), or TBPL1 instead of TBP (p-value < 0,001) were applied in triple transfections (Figure 4A). Replacing Geminin by a mutant lacking the coiled-coil domain (Gmnn m2) did not change the synergistic effect in triple transfections, indicating that it did not result from a perturbation of the cell cycle or of DNA replication. Transfection of a Geminin mutant lacking the binding domain for Brg-1 (Gmnn m3) did not reduce the activity, but resulted in a slight enhancement (Figure 4B).

Similar transfection experiments were performed comparing the non-mutated AdML promoter with mutants in the binding sites for either TBP (m1) or TIPT2 (m3). The background level achieved with single or double transfections was slightly elevated when TIPT2 and geminin vectors were transfected together (Figure 4C). Here, differences between the wild-type AdMLP reporter and reporters with a mutation in either the TIPT2-binding BRE element, or the TBP-binding TATA box were not recognizable. Double transfections did not produce a significant difference between the luciferase activities of BRE^u ^mutant or TATA box mutant and wild type AdML promoter (p-value > 0,01). Maximal luciferase activities were obtained in triple transfections using the wild-type form. Both the TATA box and BRE^u ^mutated promoters gave lower activation levels compared to the wild-type promoter (p-value < 0,001, respectively < 0,01; Figure 4C).

To investigate if TIPT2, TBP and geminin also activated a different TATA-containing promoter, the herpes virus thymidine kinase (TK) promoter was assayed, which does not contain a BRE element upstream of its TATA box. Still, the transfection results were quite similar to the AdMLP data. Single transfection of TBP, TIPT2 or geminin, as well as double transfections of TBP/TIPT2 or TBP/geminin activated expression only marginally (2–4 fold; Figure 4D). Triple transfections of TBP, TIPT2, and geminin activated the reporter very strongly (29 fold) and synergistically. This effect was TBP-specific, because it could not be obtained by adding TBPL1 (p-value < 0.01; Figure 4E).

The indicated reporter assays were realized in the presence of endogenous TIPT2, geminin and TBP, all three proteins being present in the U2OS cell line. We tried to knock-down endogenous TIPT2 using various small interfering (si) RNAs and short hairpin constructs. While TIPT2 RNA levels could be decreased more than 80%, the protein levels were not significantly affected even after several days and repeated siRNA transfection (data not shown). However, endogenous geminin levels could be knocked-down efficiently with specific siRNA molecules (Figure 4G). The experiments indicated that both single (TBP; p-value < 0,001) and double overexpressions (TIPT2/TBP; p-value < 0,001) were reduced in the absence of geminin (Figure 4F). Together, these results suggest that TIPT2 synergizes with geminin and TBP in order to activate TATA-containing promoters, both in the presence or absence of a TIPT2 binding DNA sequence element. We see significant, logic changes in the activation of TATA promoters in response to different protein combinations. It is however apparent, that we did not necessarily observe a complete abrogation, if one factor was missing, mutated, or its binding site was removed.

### Association of TIPT2 and TBPL1-TFIIA with the neurofibromin promoter

**Figure 5:**
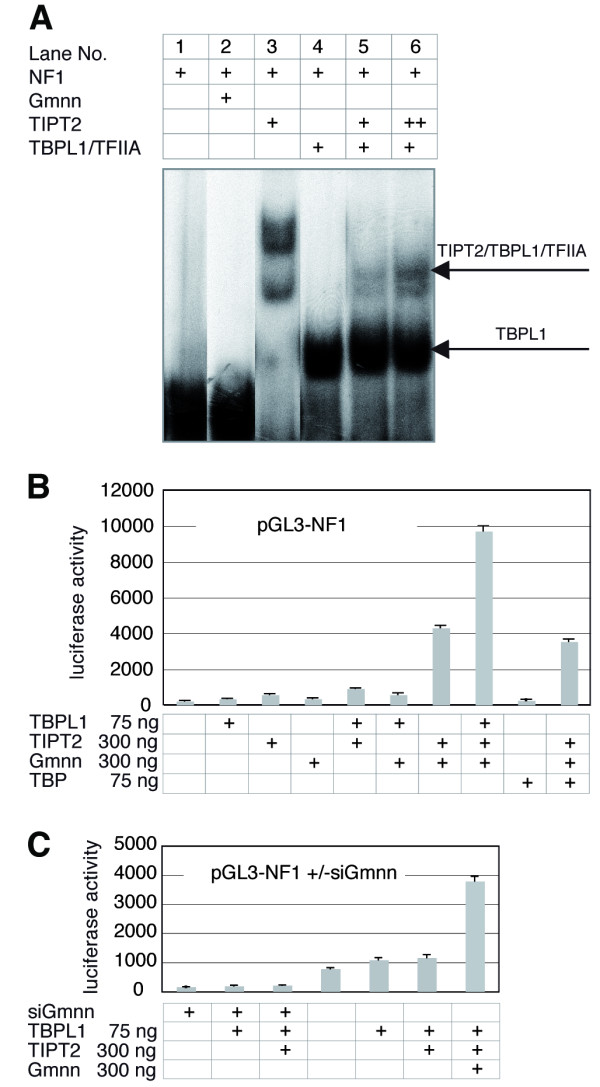
**Binding and activation of the NF1 promoter by TIPT2 and TBPL1**. (A) TIPT2 (120–240 ng) and TBPL1-TFIIA (20 ng) binding to the NF1 oligonucleotide. Geminin (120 ng) binding to the NF1 oligonucleotides. (B) Activation of the NF1 promoter by the indicated combinations of TIPT2, TBPL1 and geminin. (C) Activation of the NF1 promoter by the indicated combinations of TIPT2, TBPL1 and geminin in the presence of siRNA against geminin.

Recently, the binding of TBPL1 to a 103 bp fragment of the NF1 promoter was demonstrated *in vitro *and *in vivo *[26]. It was shown that TBPL1 binds to this promoter on two different sites and that TBPL1 shifts DNA in complex with TFIIA. We purified the TBPL1-TFIIA complex from cytoplasmic and nuclear extracts of a HeLa cell line stably overexpressing Flag tagged TBPL1 [44]. The NF1 oligonucleotide was retarded significantly by this protein fraction (Figure 5A, lane 4), while geminin did not bind to it (Figure 5A, lane 2), and TIPT2 produced bands, which however were not completely competed for by cold oligonucleotides (Figure 5A, lane 3). Incubation of increasing amounts of TIPT2 with the NF1 oligonucleotide in the presence of TBPL1-TFIIA resulted in the appearance of a novel, slower migrating band, suggesting the binding of TIPT2 and TBPL1-TFIIA on the same oligonucleotide (Figure 5A, lanes 5,6). An influence of geminin on the TIPT2/TBPL1-TFIIA/NF1 complex could not be demonstrated clearly by an electrophoretic mobility shift. Together, these data indicate that TIPT2 associates with TBPL1-TFIIA on the TATA-less NF1 promoter.

### TIPT2, TBPL1 and geminin synergize to activate the TATA-less NF1 promoter

We next analyzed whether TIPT2 was able to contribute to the activation of the NF1 promoter by employing luciferase reporter constructs with the NF1 promoter, containing multiple TBPL1 binding sites [26]. TBPL1, TIPT2 or geminin alone were not capable of activating the reporter significantly (Figure 5B). TIPT2 and geminin together produced already a significant increase (20 fold), and the additional inclusion of TBPL1 led to a very strong, synergistic activation (44 fold; Figure 5B). Replacing TBPL1 by TBP revealed that the triple combination TBP/TIPT2/geminin was not more active than just TIPT2/geminin (p-value < 0,001), indicating that NF1 activation was TBPL1-specific (Figure 5B). A knock-down of endogenous geminin decreased the activity of the NF1 reporter in the presence of exogenous TBPL1 or TIPT2/TBPL1 to levels significantly below the control background in the absence of any siRNA transfected, by a factor of about 4 (p-value < 0,01, respectively < 0,001) (Figure 5C). This result suggests that medium levels of activation in the absence of transfected geminin benefited from the presence of endogenous geminin. In summary, we show that TIPT2, TBPL1 and geminin activate the TATA-less NF1 promoter construct synergistically.

### The presence of TIPT2 and/or geminin in specific chromatin

**Figure 6:**
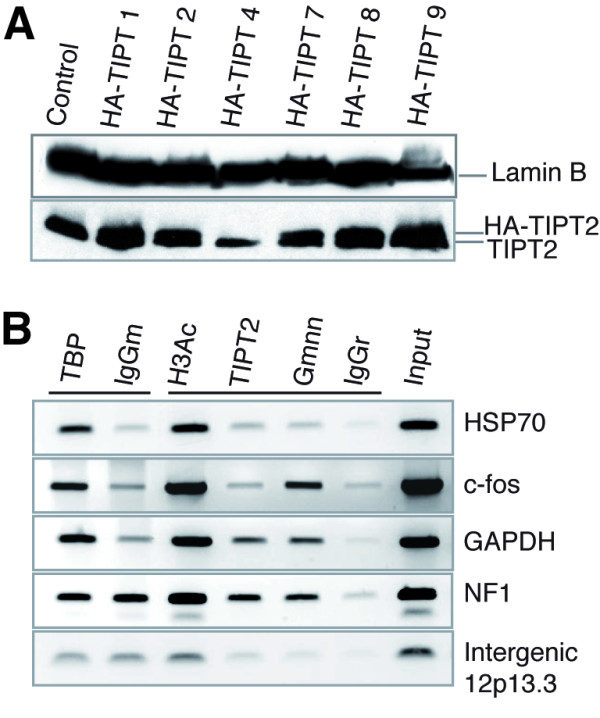
**Geminin and TIPT2 in the chromatin of TATA box-containing and TATA-less promoter regions**. ChIP assays were performed for promoter regions and the intergenic region as indicated, using the U2OS cell line stably producing HA-TIPT2 (HA-TIPT8). (A) Western blot analysis of HA-TIPT2 protein from different stably transfected clones in comparison with endogenous TIPT2 and Lamin B. (B) ChIP assay. The acetylated H3 was used as positive control. The chromatin was immunoprecipitated with normal mouse IgG (IgGm) as negative control for anti-TBP, or with normal rabbit IgG (IgGr) for anti-acetylated H3, anti-HA, and anti-geminin, respectively.

The chromatin of TBP or TBPL1 dependent genes, possessing either TATA box or TATA-less promoters, was assayed by chromatin immunoprecipitation (ChIP) using *in vivo *dual-cross-linking [45,46]. For this purpose the stable U2OS line generated, which produced HA-TIPT2 at levels similar to the endogenous protein (clone HA-TIPT8; Figure 6A) was used. The TATA box-containing regions of the investigated promoters were positive for TBP and acetylated histone H3 (Figure 6B). On the HSP70 promoter neither TIPT2 nor geminin were found. On the c-fos promoter geminin, but not TIPT2, was present in the chromatin. However, on the GAPDH promoter both TIPT2 and geminin were found. The TATA-less NF1 promoter region that includes two TBPL1 binding sites was positive for acetylated H3K9K14, geminin and TIPT2. The PCR band indicating the presence of TBP had the same intensity as for the other TATA-containing promoters, but was not enriched in comparison with mouse IgG. Thus, it had to remain inconclusive, if TBP is present in the chromatin of the NF1 promoter. These results indicate that TIPT2 and geminin are present alone or together, on the chromatin of some, but not all tested promoters.

## Discussion

Recently, information on a second isoform of TIPT (isoform 1) was independently reported [39]. These authors describe expression in male germ cells, interaction with TBPL1 (TRF2), DNA binding and association with chromatin, in line with our findings on isoform 2. However, they did not detect the widespread expression we saw on the RNA and protein level for isoform 2, although probe and antibodies would be expected to crossreact.

In our study, we describe the synergistic function of two interacting factors, TIPT2 and geminin, in the activation of transcription. TIPT2 and geminin have in common that they can interact with polycomb group members (PcGs), which are known for their function in the maintenance of gene repression [47-52]. Therefore, it came unexpected that PcGs were found together with the basal transcriptional machinery on repressed promoters [49,53]. It lead to the hypothesis that PcG proteins maintain silencing by inhibiting general transcription factor-mediated activation of transcription by interfering with the formation of the preinitiation complex [54]. Our findings suggest that TIPT2 and geminin could be involved in the transition from inactive to active transcription via association with basal transcription as well as Polycomb factors. We found both geminin and TIPT2 in the chromatin of transcribed genes. TIPT2 was significantly enriched in nucleoli, a subcellular localization shared by the bHLH transcription factor Hand1, the basal transcription factor TBPL1, by *Drosophila *testis-specific TAF homologs and, albeit as an exception, by some PcGs [40,55-57]. For the nuclear geminin, on the other hand, a nucleolar localization was not specifically described. If we observe a cooperation of these proteins in Pol II-dependent transcription, their functional interactions must occur in the nucleoplasm. Nucleoli might represent a site to keep transcription factors unavailable for gene regulatory functions, a hypothesis which may also apply to TIPT2. However, we have preliminary evidence that the nucleolar localization of TIPT2 also reflects a role in the activation of ribosomal RNA by polymerase I. In addition, our unpublished data indicate that TIPT2 nucleolar localization is regulated by phosphorylation.

Sequences flanking the core TATA box can influence the assembly of complexes, affect the basal level of transcription and the response to activators [58-60]. Our results show that TIPT2 contacts the AdMLP on a BRE site immediately upstream of the TATA box, without a need for TFIIB or TBP. In band-shift assays TIPT2/AdMLP complexes run in two or more bands (Figure 3A, lanes 2,3; 3D, lane 2; 3F, lane2). The faster migrating band represents TIPT2 associated with the BRE^u ^site of the oligonucleotide. The slower band(s) could represent more than one TIPT2 protein per oligonucleotide, with only one monomer engaged in DNA interactions. The slower migrating complex becomes more prominent than the faster complex after addition of cold oligonucleotides with BRE^u ^mutations (m2, m3, m4; Figure 3F). This might be indicative of a second DNA binding site on the TIPT2 protein interacting with the competitor.

The binding of TIPT2 to the BRE site interfered significantly with the binding of TBP to the TATA box. Both proteins on one oligonucleotide providing both binding sites were not clearly demonstratable. But some material of lower electrophoretic mobility may indicate the formation of a triple complex, containing AdMLP, TIPT2 and TBP. This disappeared upon addition of anti-TBP antibodies. ChIP experiments indicated that TBP and TIPT2 could be associated *in vivo *with chromatin in the TATA box containing promoter region of the *GAPDH *gene. While this finding does not formally prove the simultaneous presence, it appears very unlikely that the active GAPDH promoter exists in two different configurations, being occupied with either TBP or TIPT2.

Our data suggest that transcriptional activation by TIPT2 is optimal in the presence of a BRE^u ^element (AdMLP), but occurs also with a mutated (AdMLPm3) BRE^u ^element or in its absence (TK promoter). DNA binding of TIPT2 appears not to be essential, and association just by protein-protein contact seems to be sufficient. A recent bioinformatic study revealed that 24.5% of core promoters from the EPD (Eukaryotic Promoter Database: ), and 25.5% from the DBTSS (Database of Transcriptional Start Sites; ), respectively contain a BRE^u ^[4]. More precisely, 28,1% of TATA-less promoters and 11.8% of TATA-containing promoters present a BRE^u^. It is therefore conceivable that TIPT2 plays a role in the activation of transcription from many promoters.

The TIPT2 binding protein, geminin, activated transcription in several reporter assays. The simultaneous transfection of geminin and TIPT2 expression vectors boosted the activation of TATA-containing promoters. A mutant of TIPT2 that could not interact with geminin was clearly less active, again underlining the importance of the geminin/TIPT2 interaction in transcriptional activation. Our results concerning AdML promoter mutations analysis extends the previous findings that disruption of BRE^u ^decreased activated transcription [60]. Our data indicate that a similar mechanism for transcriptional activation could hold true in the case of the TATA-less NF1 promoter [61,62]. Mammalian TBPL1 does not stimulate transcription *in vitro *from TATA box-containing E4, AdML and Hsp70 promoters [24,44,63,64]. We showed here that TIPT2, TBPL1 and NF1 promoter form a complex *in vitro*. The synergistic transcriptional activation of the NF1 promoter by TBPL1, TIPT2 and geminin is similar to the synergistic activation we observed for the TATA box-containing promoters (AdML, TK) by TBP, TIPT2 and geminin. It may, however, be too simplistic to correlate exclusively the TATA box promoters with TBP, and the TATA-less promoters with TBPL1 [29].

Down-regulation of geminin levels in human U2OS cells decreased the activity obtained from activated TK and NF1 promoters, indicating that already the endogenous geminin plays a significant role in our transcriptional assays. Geminin was reported to be involved in different, transcription-related complexes as a negative regulator of transcription, either directly or indirectly. In this study we show geminin's function as transcriptional co-activator. Other studies have shown that another chromatin remodeler, the SWI/SNF complex, is able to act in a gene-dependent manner either as activator or repressor [65,66]. Our results suggest that geminin may interact with distinct protein complexes that exhibit cell-type or gene-specific functions. These interactions may decide if geminin affects transcription as a transcriptional repressor or activator.

In agreement with *in vitro *and reporter assay data, the chromatin analysis revealed geminin and TIPT2 on TATA-containing and TATA-less, active gene promoters. On the active endogenous chromatin, TIPT2 and geminin were present near the TATA box of the *GAPDH *gene, but not of the *c-fos *or the *HSP-70 *gene on a distance of two-three nucleosomes. The active TATA-less NF1 promoter is associated with geminin and TIPT2 in the region where TBPL1 was shown to bind. Our own ChIP evidence does not allow to conclude for the presence of TBP in the chromatin of the NF1 promoter in U2OS cells. However, recently the presence of TBP was described in the chromatin of the NF1 promoter in HeLa cells [29].

A genome-wide analysis of TRF2 recognition sites in *Drosophila *indicates that TRF2, a protein closely related to vertebrate TBPL1, plays a more general role than previously thought, being required for the expression of more than 1,000 genes, some involved in regulation of chromatin organization and cell growth (*Histone H1 linker *and ribosomal protein genes) [30]. This may suggest also that in mammals TBPL1 plays an important role regulating essential cell functions not only in testis, but also in other tissues. In this new perspective, our study might indicate new factors binding to both TBP and TBPL1, which might constitute co-regulatory proteins common for both core promoter corresponding complexes.

## Conclusion

In conclusion, we suggest that the two interacting proteins TIPT2 and geminin function as synergistic transcriptional regulators in contact with both the basal transcriptional machinery and chromatin factors, including members of the Polycomb complex.

## Methods

### Plasmids constructions

The primers used for this study are listed in Additional file 2. The full-length TIPT2 clone was obtained from the mouse 8.5 cDNA library used in the yeast-two-hybrid screen, and included start codon, stop codon and poly A tail [34].

To construct mouse GST-TIPT2, TIPT2 was amplified from pPC86-TIPT2, and cloned into pGEX-KT. For the HA-TIPT2 expression construct, the coding sequence was inserted into the pcDNA vector (Invitrogen) containing an intron and a HA epitope (provided by R. Lührmann). The pFlag-HA-Myc-TIPT2 (FHM-TIPT2) construct was generated by inserting a TIPT2 PCR product in frame with the triple tag into the previously described pFHM-IRESNeo vector [67]. For *in vitro *transcription/translation, SP6/T7 vectors were used or generated with the complete coding sequences for TIPT2, geminin, Scmh1 [34], Mel18, Ring1B (provided by H. Koseki), Mph2, TBPL, TBP [68], and TFIIB [69]. GFP-TBP and GFP-TBPL1 plasmids were described before, and provided by J.A. Chong [26]. The pCMV3-geminin construct was previously described [34], mutant geminin expression vectors lacking the coiled-coil (Gmnn m2), or the Brg1 binding domain (Gmnn m3), respectively, were provided by K. Kroll [37]. Reporter plasmids were based on pGL3-Basic (Promega), such as pGL3-TK, pGL3-AdMLP (position -48 to +11; Figure 3E), pGL3-AdMLPm1 (AdML promoter mutated for TATA box; Figure 3E), pGL3-AdMLPm3 (AdML promoter mutated for TIPT2 binding; Figure 3E), and pGL3-AdE4 (position -56 to +39; Figure 3E). To construct the pGL3-NF1 reporter, first the human NF1 promoter region (position -335 to +15) [26] was amplified from human genomic DNA, then cloned into pGL3-Basic, and then, fourfold-reiterated oligonucleotides (position -282 to -259) were ligated into pGL3-NF1. A plasmid encoding GST-TIPT2 with a mutation in the geminin binding site (GST-TIPT2m) was generated from GST-TIPT2 plasmid by site directed mutagenesis. GFP-15.5 K was provided by R. Lührmann.

### Protein preparations

Whole cell extract was prepared from HeLa cells using a modified RIPA buffer (50 mM Tris, pH 7.4, 1% NP-40, 0,25% Na-deoxycholate, 0.5 mM EDTA, 150 mM NaCl, 1 mM PMSF). U2OS whole cell extract was prepared using lysis buffer (20 mM Tris, pH 7.4, 150 mM NaCl, 1% NP-40, 1 mM PMSF). Testis extract was prepared from liquid nitrogen frozen testes of 10 weeks old male mice using testis lysis buffer (50 mM Tris, pH 8.0, 150 mM NaCl, 0.5% NP-40, 1 mM PMSF). Organ extracts were prepared from freshly dissected P2 mice using lysis buffer containing 20 mM Tris, pH 7.5, 150 mM NaCl, 1% NP-40, 1 mM EDTA, 1 mM DTT and protease inhibitor cocktail. TFIIA-TBPL1 complex was purified according to [44], human TBP according to [70], GST-geminin and His-geminin according to [34]. A peptide array (Jerini, Berlin) of overlapping 20-mers derived from the TIPT2 sequence was incubated with His-geminin and analyzed as described previously [34].

### Recombinant TIPT2 and anti-TIPT2 antibodies

Full-length TIPT isoform 2 and a TIPT2 version mutated in the geminin binding site were produced as N-terminal GST fusions by standard procedures. Highly pure protein preparations were obtained by affinity chromatography on Glutathione Sepharose. GST-free TIPT2 was generated by thrombin cleavage on the gel matrix, and thrombin removal by benzamidine chromatography. TIPT2 was further purified from a preparative 15% SDS-PAGE gel, and its identity was confirmed by mass spectrometry. Gel-purified TIPT2 protein was used to generate polyclonal rabbit antibodies (Bioscience, Göttingen). The crude anti-TIPT2 rabbit serum was purified by chromatography on matrix coupled GST-TIPT2.

### GST pull-down assay

Either radioactive proteins or whole cell extracts were used as preys. Radioactive binding proteins were generated by transcription/translation using the TNT SP6/T7 Coupled Reticulocyte Lysate System (Promega). For the preparation of the pull-down bait, Glutathione Sepharose-4B beads (Amersham) were pre-washed in lysis buffer (50 mM Tris pH 7.5, 500 mM NaCl, 2 mM EDTA, 5 mM DTT, 10% glycerol, 1 mM PMSF, Complete-EDTA protease inhibitors). For coupling, GST-TIPT2, GST-TIPT2m, GST-geminin, or GST, respectively, were incubated with Glutathione Sepharose-4B beads in binding buffer (20 mM Tris pH 7.5, 100 mM NaCl, 1 mM EDTA, 0,1% NP-40, 1 mM PMSF, protease inhibitors). GST protein-loaded beads were washed twice, incubated with radioactively labelled protein or protein lysates (from U2OS cells or adult mouse testis) for 1 hour at 4°C in 500 μl of binding buffer, washed twice in binding buffer, and then twice in washing buffer containing 150 mM NaCl. Proteins were eluted in 40 μl SDS loading buffer at 95°C. 20 μl of the eluted protein were electrophoresed, in parallel to an input lane containing 20% of the original radioactive protein preparation, 4% of total U2OS whole cell extract or 10% of testis extract.

### Immunoprecipitation

Immunoprecipitations were performed using the anti-HA affinity matrix (Roche). U2OS and stable, HA-TIPT2 producing cells (HA-TIPT8) were used to prepare the protein extracts and to immunoprecipitate HA-TIPT2 and endogenous TBP. The cells were washed with cold PBS, and lysed with lysis buffer (50 mM Tris-HCl, pH 7.4, 120 mM NaCl, 1 mM EDTA, 1 mM EGTA, and 1% NP-40, 10 mM β-glycerol-P, 5 mM K_2_HPO_4_) by passage through the needles and incubating for 1 hour at 4°C. The extracts were centrifuged, and the supernatants were incubated with identical amounts of anti-HA agarose beads for several hours at 4°C. After washing (50 mM Tris HCl, pH 7.4, 150 mM NaCl, 0.1% NP-40), the complexes were eluted by boiling with 2× sample buffer (125 mM Tris HCl, pH 6.8, 4%SDS, 20% (v/v) glycerol, 0,004% bromphenol blue). 2.5% of the inputs and 50% from the eluates were electrophoresed.

### Antibodies

The following primary antibodies were used: rabbit anti-TIPT2, raised against *E.coli-*expressed gel-purified full-length TIPT2; rabbit anti-geminin (sc-13015, Santa Cruz); mouse anti-TBP (3G3; gift from I. Davidson); mouse anti-TBP (Mab-TBPCSH-100, Diagenode); mouse anti-TBPL1 (2A1, provided by I. Davidson); rabbit anti-HA (ab9110, Abcam); rat monoclonal anti-HA (clone 3F10, 1867431, Roche); goat anti-Lamin B (sc-6216, Santa Cruz); mouse anti-GST (71097–3, Novagen); rabbit anti-acetyl histone H3 (06–599, Upstate); rabbit IgG (12–370, Upstate); mouse IgG (12–371, Upstate); mouse anti-NPM (32–5200, Zymed).

The following secondary antibodies were used: HRP-conjugated goat anti-rat IgG (112-035-143, Jackson ImmunoResearch); HRP-conjugated rabbit anti-goat IgG (ab6741-1, Abcam); HRP-conjugated goat anti-rabbit IgG (211–1303, Rockland); HRP-conjugated goat anti-mouse IgG (115-035-003, Jackson ImmunoResearch); Alexa Fluor 594 and 488 goat anti-rabbit IgG (A11012, A11008, Molecular Probes); Alexa Fluor 594 and 488 goat anti-mouse IgG (A-11005, A-11001, Molecular Probes); Alexa Fluor 594 chicken anti-rat IgG (A21471, Molecular Probes).

### Electrophoretic mobility shift assays

*In vitro *interactions of human TBP, human TBPL1 complexed with TFIIA, recombinant GST-TIPT2 and His-geminin proteins with various promoter sequences were tested. The following, double-stranded oligonucleotides (see Additional file 2 and Figure 3E for sequences) were investigated: AdMLP oligonucleotide (position -40 to -16) [71], AdMLPm1-m9, NF1 oligonucleotide (position -288 to -253) [26,72], AdE4 oligonucleotide [43]. Binding reactions were performed at a final concentration of 5.5 nM labeled probe, no corrections for cpm were made. Competition by non-specific oligonucleotides in Figure 3A was performed at 0.03 μM poly(dI-dC), 0.33 μM poly(dG-dC), 0.08 μM or 0.4 μM double-stranded cold AdMLP. 0.1 μl of mouse anti-TBP (3G3) or mouse anti-GST antibodies (Novagen) were applied in the last seven minutes of the binding reactions (Figure 3B, G). For the competition experiments between AdMLP and TIPT2 by mutated versions of AdMLP double-stranded, unlabeled oligonucleotides were added at a final concentration of 1.6 μM or 5 μM, respectively, in the presence of 6 ng GST-TIPT2 (Figure 3F). Radiolabeled probe was added 15 min after starting the reaction.

Different binding reactions and electrophoresis conditions were performed for TBP or TBPL1, respectively. TBP binding reactions were performed in a final volume of 15 μl by mixing 3 μl 5× buffer (60 mM HEPES/KOH, pH 7.6, 300 mM KCl, 15 mM dithiothreitol, 1 mM EDTA, 10% polyethylene glycol 8000 (w/v), 20 mM MgCl_2_, 40% glycerol, 0.1% NP-40, 66.65 μg/ml BSA) with poly(dG-dC) (0.08 μM final concentration), 6 ng TBP, and labeled oligonucleotides (5.5 nM final concentration) [73].

The TBPL1 binding reactions were performed in a final volume of 15 μl by mixing 5 μl 3× buffer (60 mM HEPES/KOH, pH 7.6, 180 mM KCl, 9 mM dithiothreitol, 60% glycerol, 60 μg/ml BSA) with 200 ng BSA, poly(dG-dC) (0.03 μM final concentration), and 20 ng TBPL1-TFIIA complex [26]. For checking the binding of both TBP and TIPT2 to AdMLP oligonucleotides, the proteins were added simultaneously to the binding reactions. The reactions were incubated at 30°C for 45 minutes. For the electrophoresis 4% polyacrylamide mini-gels (59:1 mono:bisacrylamide ratio, 5% glycerol, 1 mM DTT) were polymerized for minimum 1 hour and prerun for 45 minutes at 200 V and 4°C. For TBP bandshifts the gel was electrophoresed in 0.5× Tris-borate-EDTA (TBE) buffer supplemented with 5% glycerol [74] and 0.02% NP-40 for 20 minutes at 200 V at 4°C. The TBPL1 gel shift was electrophoresed in 0.5× TBE buffer for 16 minutes at 400 V and 4°C.

### Cell culture and ChIP assay

For the stable production of tagged TIPT2 version, U2OS cells were transfected with HA-TIPT2 or FHM-TIPT2 expression constructs containing a Neomycin gene cassette. Clones were selected with 500 μg/ml neomycin (G418), and analyzed for HA-TIPT2 or FHM-TIPT2 expression.

1.5 × 10^7 ^HA-TIPT2 U2OS cells were used for 6 ChIP reactions. Disuccinimidyl glutarate (20593, Pierce) was applied to the washed cells at a final concentration of 2 mM in PBS pH 8.0, supplemented with 1 mM MgCl_2_for 30 min at RT. The cells were washed, 1.42% formaldehyde in PBS was applied for 20 min, and the reaction was stopped with glycine. Subsequently, cells were lysed in 1% SDS buffer, and the chromatin shearing was performed using the Bioruptor XL sonicator (Diagenode) at 4°C to obtain 200–500 bp DNA fragment size. Antibodies were used for immunoprecipitation of precleared chromatin. The complexes were collected using either protein G or protein A sepharose beads (16–201, 16–157, Upstate), the beads were washed, and the complexes were eluted. The crosslinking was reversed for 6 hours at 65°C. After RNase and proteinase K treatment the DNA was purified using the QIAquick PCR purification kit (Qiagen), and PCR reactions were performed. The primers used in the PCR reactions are described in Additional file 2.

### Luciferase assay

U2OS cells were transfected in 24-well plates with the Fugene6 (Roche). After 36 h cells were processed for analysis with the Dual-Luciferase Reporter Assay System (Promega). A Renilla reporter (pRL-CMV, pRL-SV40, Promega) as internal standard was not included, since it turned out to be variably affected by introduced transcription factors. Each transfection experiment was performed in four parallel wells containing equal numbers of cells. The mean values, standard deviations and p-values were calculated. The p-values were obtained using a paired t-test. In text p-value < 0.01 indicates significant differences, and highly significant differences are presented as p-value < 0,001. The total amount of DNA per well was kept constant at 1425 ng by the addition of CMV-GFP. The reporter-TBP/TBPL1 expression vector ratio was always 10:1. Transfection experiments were repeated three times.

### Geminin knock-down

U2OS cells were transfected in 24-well plates with siGENOME SMARTpool human geminin (M-003270-01, Thermo Fisher Scientific) to obtain a 100 nM final concentration, using Dharmafect1 transfection reagent (Thermo Fisher Scientific). The control cells were treated in a similar way, except for the siRNA that was replaced by the siRNA dilution buffer (Roche). After 6 hours the cells were transfected with reporter and expression plasmids. After 24 h the cells were processed for analysis with the Dual-Luciferase Reporter Assay System (Promega).

## Authors' contributions

MEP carried out the biochemical and cell biological studies, participated in the development of the concept and drafted the manuscript. LL identified and provided the original cDNA clone. MT contributed essential protein purifications, and participated in the evaluation and discussion of the data. MK conceived of the study, and participated in its design and coordination, and helped to draft the manuscript. All authors read and approved the final manuscript.

## Supplementary Material

Additional file 1 **Subcellular localization of TIPT2 in cultured cells**. This file contains a supplemental immunohistochemical analysis of TIPT2 expression in cultured cells. Click here for file

Additional file 2 **Table of oligonucleotide sequences**. This table supplies the sequences of all oligonucleotides used in this study. Click here for file
