# Supplementary material for: TIPT2 and geminin interact with basal transcription factors to synergize in transcriptional regulation
Source: BMC Biochem. 2009 Jun 10;10:16. doi: 10.1186/1471-2091-10-16 (PMC2702275; doi:10.1186/1471-2091-10-16)
Supplement: Additional file 1 — Subcellular localization of TIPT2 in cultured cells. This file contains a supplemental immunohistochemical analysis of TIPT2 expression in cultured cells. [file 1471-2091-10-16-S1.doc]

**
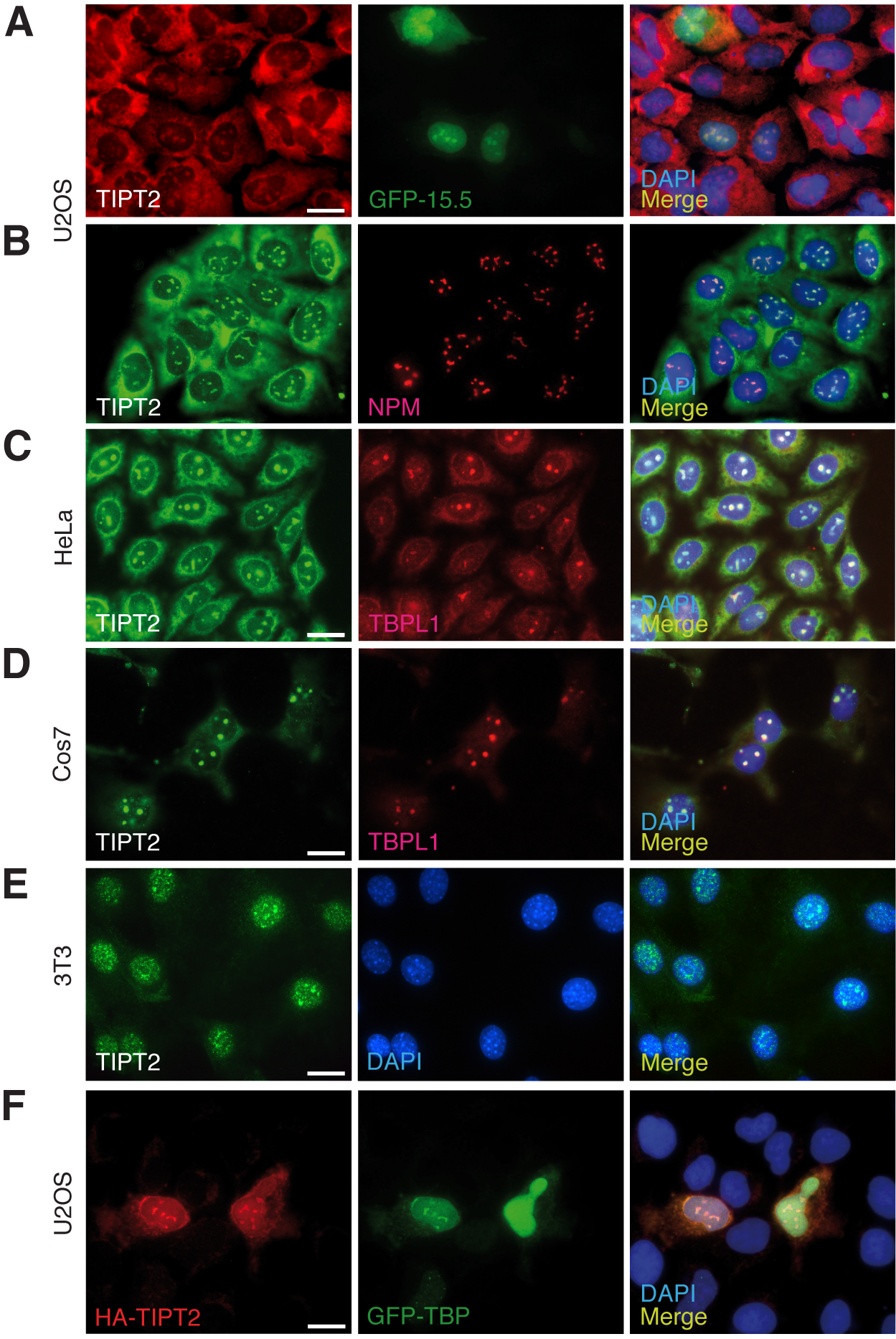
**

**Subcellular localization of TIPT2 in cultured cells.**

(A) U2OS cells were transfected with an expression vector encoding the nucleolar marker protein 15.5K fused to GFP. Immunohistochemistry with anti-TIPT2 antibodies indicates co-localization in nucleoli and cytoplasmic presence of TIPT2. (B) U2OS cells were stained simultaneously with anti-TIPT2 and anti-nucleophosmin (NPM) antibodies. Co-localization was observed in the nucleolus. TIPT2 was detected additionally in the cytoplasm. (C) HeLa or (D) Cos7 cells, respectively, were stained simultaneously with anti-TIPT2 and anti-TBPL1 antibodies. Co-localization was observed in the nucleolus, TIPT2 was detected additionally in the cytoplasm, and less pronounced in the nucleoplasm. (E) Nuclear and nucleolar expression of TIPT2 in murine 3T3 cells. (F) Nuclear and nucleolar expression of transfected HA-TIPT2 and GFP-TBP U2OS cells. Bar, 20 μm.
