# Supplementary material for: TIPT2 and geminin interact with basal transcription factors to synergize in transcriptional regulation
Source: BMC Biochem. 2009 Jun 10;10:16. doi: 10.1186/1471-2091-10-16 (PMC2702275; doi:10.1186/1471-2091-10-16)
Supplement: Additional file 2 — Table of oligonucleotide sequences. This table supplies the sequences of all oligonucleotides used in this study. [file 1471-2091-10-16-S2.doc]

**Supplementary Table 1: Oligonucleotide sequences**

**Primers for TIPT probe**

5’ – AAGATCCCCGGGATGGCGACGCCCCTTGGGTG – 3’

5’ – AAGATCAAGCTTGGTGGCGGCCGTTACTTACTT – 3’

**Primers for GST-TIPT**

5’ – AAGATCCTCGAGATGGCGACGCCCCTTGGGTG – 3’

5’ – AAGATCAAGCTTGGTGGCGGCCGTTACTTACTT – 3’

**Primers for HA-TIPT**

5’ – AAGATCAGATCTTAATGGCGACGCCCCTTGGGT – 3’

5’ – AAGATCGCGGCCGCAGCAGTTTATTTCTCCCTGGAA – 3’

**Primers for FHM-TIPT**

5’ - AAGATCGCTAGCATGGCGACGCCCCTTGGGTG – 3’

5’ - AAGATCAGATCTGGTGGCGGCCGTTACTTACTT – 3’

**Primers for GST-TIPTm and pCMV-TIPTm**

5’ – GTGCTGGATCAGGATCGTGATAAAGCCACAGGAATGC – 3’

5’ – GCATTCCTGTCGCTTTATCACGATCCTGATCCAGCCAC – 3’

**Primers for pSP64-Mph2**

5’ – AAGATCAAGCTTATGACCTCAGGGAACGGAAAC – 3’

5’ – AAGATCTCTAGATGATGTTCATGGCGCTCATTA – 3’

**Primers for pSP64-TBPL1**

5’ – AAGATCGTCGACATGGATGCAGACAGTGATGTTGC – 3’

5’ – AAGATCTCTAGAGAGTTATAAAATCTCCTTCCTGC – 3’

**Oligonucleotides for pGL3-AdMLP**

5’ - TCGAGTGTTCCTGAAGGGGGGCTATAAAAGGGGGTGGGGGCGCGTTCGTCCTCACTCTCTTCCGA – 3’

5’ - AGCTTCGGAAGAGAGTGAGGACGAACGCGCCCCCACCCCCTTTTATAGCCCCCCTTCAGGAACAC – 3’

**Oligonucleotides for pGL3-AdMLPm1**

5’ - TCGAGTGTTCCTGAAGGGGGGCTGTAAAAGGGGGTGGGGGCGCGTTC GTCCTCACTCTCTTCCGA – 3’

5’ - AGCTTCGGAAGAGAGTGAGGACGAACGCGCCCCCACCCCCTTTTACAGCCCCCCTTCAGGAACAC – 3’

**Oligonucleotides for pGL3-AdMLPm3**

5’ - TCGAGTGTTCCTGAAGGTTGGCTGTAAAAGGGGGTGGGGGCGCGTTCGTCCTCACTCTCTTCCGA – 3’

5’ - AGCTTCGGAAGAGAGTGAGGACGAACGCGCCCCCACCCCCTTTTACAGCCAACCTTCAGGAACAC – 3’

**Oligonucleotides for pGL3-AdE4**

5’ - TCGAGCGTTACGTCATTTTTTAGTCCTATATATACTCGCTCTGTACTTGGCCCTTTTTACACTGTGACTGATTGAGCTGGTGCCGTGTCGAGTGGTGA – 3’

5’ - AGCTTCACCACTCGACACGGCACCAGCTCAATCAGTCACAGTGTAAAAAGGGCCAAGTACAGAGCGAGTATATATAGGACTAAAAA ATGACGTAACG C – 3’

**Primers for pGL3-NF1**

5’ – AAGATCCTCGAGGCTACGAAGAACCTGAAAATCGGA – 3’

5’ – AAGATCAAGCTTTGAAAAAGCGATCCTCCTGGAGG – 3’

**Oligonucleotides for pGL3-NF1**

5’ - CTAAGCTGAGAGCACAGCCTCCCCATAAGCTGAGAGCACAGCCTCCCCATAAGCTGAGAGCACAGCCTCCCCATAAGCTGAGAGCACAGCCTCCCCAG – 3’

5’ - CTAGCTGGGGAGGCTGTGCTCTCAGCTTATGGGGAGGCTGTGCTCTCAGCTTATGGGGAGGCTGTGCTCTCAGCTTATGGGGAGGCTGTGCTCTCAGCTTAGGTAC – 3’

**Oligonucleotides for NF1 gel shift assay**

5’ – AGAGCTTAAGCTGAGAGCACAGCCTCCCCAGGAGAT– 3’

5’ – ATCTCCTGGGGAGGCTGTGCTCTCAGCTTAAGCTCT– 3’

**Human GAPDH promoter (Upstate) primers for ChIP**

5’ – TACTAGCGGTTTTACGGGCG – 3’

5’ – TCGAACAGGAGGAGCAGAGAGCGA – 3’

**Human c-fos primers for ChIP**

5’ – CGTGGTTGAGCCCGTGACGTT– 3’

5’ – TGGCGGTTAGGCAAAGCCGG– 3’

**Human HSP70 primers for ChIP**

5’ – AGCCTCATCGAGCTCGGTGATTGG– 3’

5’ – AAGGTAGTGGACTGTCGCAGCAGC– 3’

**Human NF1 primers for ChIP**

5’ – ATCGGAGGTCGTGTACCTTAT – 3’

5’ – TTGGATTGCTCCCGGAGTTGG – 3’

**Human genomic region (Upstate) primers for ChIP**

5’ – ATGGTTGCCACTGGGGATCT– 3’

5’ – TGCCAAAGCCTAGGGGAAGA– 3’
